# Phylogenetics of *Ogyges* Kaup and the biogeography of Nuclear Central America (Coleoptera, Passalidae)

**DOI:** 10.3897/zookeys.737.20741

**Published:** 2018-02-13

**Authors:** Enio B. Cano, Jack C. Schuster, Juan J. Morrone

**Affiliations:** 1 Museo de Zoología "Alfonso L. Herrera", Departamento de Biología Evolutiva, Facultad de Ciencias, Universidad Nacional Autónoma de México (UNAM), Apdo. postal 70-399, 04510 Mexico City, Mexico; 2 Universidad del Valle de Guatemala, Apartado Postal 82, 01901 Guatemala, Guatemala; 3 Museo de Historia Natural, Escuela de Biología, Universidad de San Carlos de Guatemala, Calle Mariscal Cruz, 1-56, zona 10, Guatemala, Guatemala

**Keywords:** *Proculejus*, *Proculus*, *Oileus*, cloud forest, Mesoamerica

## Abstract

A phylogenetic morphological analysis of the genus *Ogyges* Kaup, distributed in Nuclear Central America, from Chiapas, Mexico, to northwestern Nicaragua was undertaken. Five species of *Proculejus* Kaup, distributed north of the Isthmus of Tehuantepec in Mexico, were selected as outgroup. *Ogyges* was recovered as monophyletic with three species groups: *championi*, *laevissimus*, and *crassulus*. Each species group shows a distinct, generally allopatric distribution. The *O. championi* species group, with ten species, is distributed in the Maya block, more specifically in the mountainous system north of the Motozintla-Comaltitlán fault in Chiapas, and north of the dry valleys of the Cuilco and Motagua rivers in Guatemala. The two remaining species groups are distributed in the Chortis block. The *O. laevissimus* species group, including seven species, ranges mostly along the Pacific Volcanic Chain from Guatemala to El Salvador, and from southeastern Honduras to the northwestern area of Nicaragua. The *O. crassulus* species group, with ten species, is distributed from northeastern Guatemala (Merendón) to northern Honduras. The Isthmus of Tehuantepec in Mexico, the Motagua-Cuilco and Motozintla-Comaltitlán sutures zones in Chiapas and Guatemala, the lowland valleys of Colón and Comalí rivers between Nicaragua and Honduras (or, perhaps, the northern suture of the Siuna Terrane in Nicaragua), the Guayape fault system in Honduras, and the intricate dry valleys of Ulúa-Chamelecón-Olancho in Honduras, are hypothesized to have acted as barriers that affected the geographical distribution of *Ogyges*, as well as probably other montane organisms.

## Introduction

Nuclear Central America (Schuchert 1935), the mountainous region comprising Chiapas (Mexico), Guatemala, Belize, El Salvador, Honduras, and northern Nicaragua, is characterized by several large and high mountain and volcanic ranges reaching an elevation of 4222 m, separated by deep and dry valleys, with the consequent isolation and independent evolution of populations. With few exceptions (e.g. Wake and Lynch 1976, Johnson 1989, Campbell and Frost 1993, Townsend 2014, Pérez-Consuegra and Vásquez-Domínguez 2015, Hofmann and Townsend 2017), its biotic relevance has been overlooked by biogeographers, and phylogenetic analyses of taxa endemic to this area are scarce. The biota has been studied as part of North American, Neotropical, Mexican, Middle American, Mesoamerican or Central American regions, and often is considered a “mixture” of North and South American elements, obscuring the in situ diversification of supraspecific taxa. Nuclear Central America is particularly speciose in endemic taxa such as plethodontid salamanders (Campbell et al. 2010, Townsend 2014, Rovito et al. 2015), cricetid mice (Conroy et al. 2001; Gutiérrez-García and Vásquez-Domínguez 2012, 2013; Ordóñez-Garza et al. 2014; Pérez-Consuegra and Vásquez-Domínguez 2015), squamates (Campbell and Frost 1993, Campbell and Brodie 1999, Castoe et al. 2003, Hasbún et al. 2005, Townsend et al. 2013, Hofmann and Townsend 2017) and beetles (Schuster 1993, Micó et al. 2006, Cano 2014, Sokolov and Kavanaugh 2014).

*Ogyges* Kaup, a flightless genus of the saproxylophagous family Passalidae, consists of 25 described species restricted to the cloud forests of Chiapas to northern Nicaragua (Cano 2014, 2017). A phylogenetic morphological analysis recovered *Ogyges* as monophyletic and closely related to the also flightless genera *Proculus* and *Proculejus* (Boucher 2006). Cano (2014) showed that the shape of the suprainternal mandibular tooth represents an exclusive synapomorphy for the species of *Ogyges*. *Proculus* includes seven species, all gigantic (50–80 mm) and with many autapomorphies, distributed in Nuclear Central America from the Chimalapas region, Oaxaca (Delgado and Mora-Aguilar 2014), to northern Honduras, being also probably distributed in the Chocó area in Colombia (Schuster et al. 2003). *Proculejus* is found in Mexico north of the Isthmus of Tehuantepec (Reyes-Castillo 1970, Boucher 2006), it includes at least 10 species and is rather similar to *Ogyges*, except for the presence of a frontoclypeal suture and a different form of suprainternal mandibular tooth. The recent discovery of a new Honduran species with a clearly marked suture on the frontoclypeus makes it difficult to place it in either genus.

A phylogenetic morphological analysis was undertaken to test the monophyly of *Ogyges*, including the new Honduran species, and using *Oileus sargi* (Kaup) and five species of *Proculejus* as outgroups. Based on the resulting cladogram, we conducted a biogeographical analysis to describe the areas of distribution and possible barriers, applying the results of the analysis of the biogeography of Nuclear Central America in an evolutionary framework.

## Methods

1073 adult specimens were examined (see Appendix 1), belonging to 33 species, deposited in the following collections:

**BMNH** The Natural History Museum, London, Great Britain.

**IBUNAM** Instituto de Biología, Universidad Nacional Autónoma de México, México City, México.

**INECOL** Instituto de Ecología, Xalapa, Veracruz, México.

**JYC** Jiichiro Yoshimoto, private collection, Guatemala City, Guatemala.

**MNHN** Muséum national d’Histoire naturelle, Paris, France.

**RC** Ronald D. Cave, private collection, Fort Pierce, Florida, USA.

**USAC** Universidad de San Carlos de Guatemala, Guatemala City, Guatemala.

**UVGC** Universidad del Valle de Guatemala, Guatemala City, Guatemala.

For terminology of the head Boucher (2006) is followed, which is based on well-supported homologies; however, instead of the terms central tubercle, orbital canthus, and inner tubercles, we use center horn, ocular canthus, and internal tubercles, respectively. For terminology of the rest of the body we follow Reyes-Castillo (1970). Measurements were taken with a digital vernier caliper except for the diameter of punctures and the antennal and femoral proportions, which were taken with an ocular micrometer in a Wild Heerbrugg M3B stereomicroscope. Total length was measured from the tip of the open mandibles to the terminal tip of the elytra. Drawings were made using a drawing tube in a Wild Heerbrugg M3B stereomicroscope. Images were taken with a Nikon D5100 camera with macro lens, except those of teeth of mandible, taken with a camera DP12 adapted to a SZX12 Olympus stereomicroscope. All images were processed with the Microsoft Digital Image Pro software.

### Outgroup selection

Although Boucher (2006: 346, 364) recovered *Proculus* as the sister group of *Ogyges*, he also encountered more than 20 autapomorphies (i.e. uninformative characters) in *Proculus*. *Proculus*, the giant passalid beetles, have more autapomorphies than any known passalid, perhaps associated with its greater size. According to Maddison et al. (1984) and Nixon and Carpenter (1994), plesiomorphic-synapomorphic states should be estimated from the outgroup, however, as Lyons-Weiler et al. (1998) state, if rates of evolutionary change vary among lineages, the sister taxon (as apparently occurs in *Proculus*) may not have the shortest evolutionary distance to the ingroup, reducing the chance that it is the optimal candidate for estimating the ingroup. In addition, outgroup choice can affect ingroup topology, even for nodes far removed from the presumed root placement (Milinkovitch and Lyons-Weiler 1998, Tarrio et al. 2000). On the other hand, the suprainternal teeth of mandibles of *Ogyges* and several species of *Proculejus* seem to be very similar, suggesting common ancestry and the possibility that both genera can be merged. For these reasons, and, in order to evaluate the monophyly of *Ogyges*, we selected five species of *Proculejus* as the outgroup to infer and select the synapomorphies and plesiomorphies of *Ogyges*, rejecting *Proculus* as a second outgroup. Additionally, due to the homoplasy involved in flightlessness, we selected the flying species *Oileus sargi* Kaup to root the resulting cladograms.

### Character analysis

A total of 53 morphological characters was used, including both external structures (48) and male genitalia (5). The distribution of character states is shown in Table 1. All multistate characters (1, 2, 7, 8, 14, 17, 19, 22, 26, 30, 32, 33, 34, 36, 39, 40, 45, and 52) were treated as non-additive. Inapplicable characters, those that describe variation with respect to the shape of some feature that is entirely absent in some taxon (Harris et al. 2003: 249), were avoided, except for characters 1, 9 and 46.

**Table 1.** Data matrix. Polymorphic states [01] are indicated by a hash symbol (#) and states [12] by a et symbol (&); inapplicable characters are indicated by a hyphen (-), and missing characters by a question mark (?). Characters are coded from 0 to 52.

											1	1	1	1	1	1	1	1	1	1	2	2	2	2	2	2	2	2	2	2	3	3	3	3	3	3	3	3	3	3	4	4	4	4	4	4	4	4	4	4	5	5	5
	0	1	2	3	4	5	6	7	8	9	0	1	2	3	4	5	6	7	8	9	0	1	2	3	4	5	6	7	8	9	0	1	2	3	4	5	6	7	8	9	0	1	2	3	4	5	6	7	8	9	0	1	2
*Oileus sargi*	0	-	0	0	0	1	1	0	0	-	0	0	1	0	0	0	0	1	0	0	0	0	0	0	1	0	0	0	1	0	2	0	0	0	0	0	2	0	1	0	0	0	0	0	0	2	-	0	0	0	0	0	0
*P. nudicostis*	0	-	1	0	0	0	1	0	2	0	0	0	1	0	0	0	0	0	0	0	0	0	0	0	1	0	1	0	1	0	0	1	0	1	0	1	2	1	0	1	3	0	0	0	1	0	-	0	0	0	0	1	1
*P. pubicostis*	0	-	1	0	0	0	1	0	2	0	0	0	1	0	0	0	0	0	0	1	1	1	1	0	1	0	1	0	0	0	1	1	0	1	0	1	1	0	0	1	3	0	0	1	0	1	1	0	0	0	0	0	0
*P. hirtus*	0	-	1	0	0	0	1	1	1	0	0	0	0	0	0	0	0	0	0	1	1	1	1	0	1	1	1	0	0	0	1	1	0	1	2	1	1	0	0	1	3	0	0	1	0	1	0	0	0	0	0	0	0
*P. brevis*	0	-	1	0	0	0	1	0	1	0	0	1	0	0	0	0	0	0	0	0	1	1	1	0	0	0	1	0	0	0	1	1	0	1	0	1	1	1	0	1	3	0	0	1	0	1	1	0	0	0	0	1	0
*P. sartorii*	0	-	1	0	0	0	0	1	1	0	0	0	0	0	0	2	0	0	0	1	1	1	1	0	1	0	1	0	0	0	1	1	0	1	0	1	1	1	0	1	3	0	0	1	0	1	0	0	0	0	0	0	0
*O. laevissimus*	1	0	2	0	1	1	1	0	2	1	0	0	0	0	0	0	0	2	1	2	1	1	2	0	0	1	2	1	1	0	1	0	0	1	0	0	0	0	0	2	1	0	0	1	1	2	-	1	1	1	0	0	1
*O. hondurensis*	1	0	2	0	1	0	1	0	2	1	0	0	0	0	0	0	0	2	1	2	1	1	2	0	0	1	2	1	1	0	1	0	1	1	0	0	0	0	0	2	1	0	0	1	1	2	-	1	1	1	0	0	1
*O. politus*	1	0	2	0	1	0	1	0	2	1	0	1	0	0	0	0	0	2	1	2	1	1	2	0	0	1	2	1	1	0	1	0	1	1	0	0	0	0	0	2	1	0	0	1	1	2	-	1	1	1	0	0	1
*O. championi*	1	2	1	1	0	0	0	0	1	0	0	1	0	0	2	1	0	0	0	1	1	1	2	0	1	1	2	1	1	1	2	0	0	2	0	0	1	0	1	0	2	0	0	1	1	2	-	0	0	0	0	0	0
*Ogyges* sp. n.1	1	2	1	1	0	0	0	1	1	0	0	1	0	0	2	1	0	0	0	1	1	1	2	0	1	1	2	1	1	1	2	0	0	2	0	0	1	0	1	0	2	0	0	1	1	2	-	0	0	0	0	0	0
*O. kekchii*	1	2	1	1	0	0	0	1	1	0	0	1	0	0	2	1	0	0	0	1	1	1	2	0	1	1	2	1	1	1	2	0	0	2	0	0	1	0	1	0	2	0	0	1	1	2	-	0	0	0	0	0	2
*O. furcillatus*	1	2	1	0	0	0	0	0	1	0	0	0	0	0	0	2	1	1	0	1	1	1	2	0	0	1	2	1	1	1	2	0	0	2	0	0	2	0	1	0	0	0	0	1	1	2	-	1	0	1	0	0	0
*O. cakchiqueli*	1	2	1	0	0	0	0	2	1	0	1	0	0	0	2	2	1	1	0	1	1	1	2	0	0	1	2	1	1	1	1	0	0	2	0	0	1	0	1	0	0	0	0	1	1	2	-	1	0	1	0	0	0
*O. coxchicopi*	1	2	1	1	0	0	0	1	1	0	0	1	0	0	&	2	1	0	0	1	1	1	2	1	0	1	2	1	1	1	1	0	0	2	0	0	1	0	1	0	0	0	0	1	1	2	-	1	0	1	0	0	0
*O. quichensis*	1	2	1	1	0	#	0	2	1	0	#	1	1	0	2	2	0	0	0	2	1	1	2	0	0	1	2	1	1	0	1	0	0	2	0	0	2	0	1	0	0	0	0	1	1	2	-	1	0	1	0	0	1
*O. tzutuhili*	1	2	1	0	0	0	0	2	1	0	0	1	0	0	0	1	1	2	1	1	1	1	2	0	0	1	2	1	1	1	1	0	0	2	0	0	2	0	1	0	2	0	0	1	1	2	-	1	0	1	0	0	1
*O. marilucasae*	1	2	1	0	0	0	0	2	1	0	1	0	0	0	2	1	0	2	1	1	1	1	2	0	1	1	2	1	1	1	1	0	0	2	0	0	2	0	1	0	0	0	0	1	1	2	-	1	1	0	0	0	1
*O. menchuae*	1	2	1	1	0	0	0	1	1	0	0	1	0	0	2	1	1	0	0	1	1	1	2	0	1	1	2	1	1	1	2	0	0	2	0	0	1	0	1	0	0	0	0	1	1	2	-	0	0	0	0	0	0
*O. crassulus*	1	2	2	0	0	1	0	0	2	1	0	0	1	0	1	1	1	1	0	0	1	1	2	1	1	0	2	1	1	1	2	0	0	2	1	0	1	0	1	1	3	1	1	1	1	2	-	1	0	0	0	0	0
*O. aluxi*	1	2	2	0	0	1	0	0	2	1	0	0	1	1	1	1	0	1	1	0	1	1	2	0	1	0	2	1	1	1	2	0	0	2	1	0	1	0	1	1	3	1	1	1	1	2	-	0	0	1	0	0	0
*O. monzoni*	1	2	2	0	0	1	0	0	2	1	0	0	1	0	1	1	0	0	0	0	1	1	2	1	1	0	2	1	1	1	2	0	0	2	1	0	1	0	1	1	3	1	1	1	1	2	-	0	0	0	0	0	0
*O. llama*	1	1	2	0	0	1	0	0	2	1	0	0	1	0	1	1	1	0	0	1	1	1	2	1	1	0	2	1	1	1	2	0	0	2	1	0	2	0	1	1	3	1	1	1	1	2	-	0	0	0	0	0	2
*O. laurae*	1	0	2	0	0	1	0	0	2	1	0	0	1	1	1	1	1	0	0	0	1	1	2	1	1	0	2	1	1	1	2	0	0	2	1	0	1	0	1	1	3	1	1	1	1	2	-	0	0	0	0	0	2
*Ogyges* sp. n.2	0	-	2	0	0	1	0	0	2	1	0	0	1	1	1	2	1	1	0	1	1	1	2	1	1	0	2	1	1	1	2	0	0	2	1	0	2	0	1	1	3	1	1	1	1	2	-	0	0	0	0	0	0
*O. adamsi*	1	0	2	0	1	1	1	0	2	1	0	1	1	0	0	1	1	2	1	2	1	1	2	0	0	1	2	1	1	0	1	0	2	1	0	0	0	0	#	1	0	0	0	1	1	2	-	1	1	0	1	0	1
*O. handali*	1	0	2	0	0	1	1	0	2	1	0	1	1	0	0	1	1	2	1	2	1	1	2	0	0	0	2	1	1	0	0	0	2	1	0	0	0	0	0	1	3	0	0	1	1	2	-	1	1	1	1	0	0
*O. sandinoi*	1	0	2	0	0	0	1	0	2	1	0	0	1	0	0	2	1	2	1	2	1	1	2	0	0	0	2	1	1	1	1	0	0	1	0	0	0	0	1	1	3	0	0	1	1	2	-	1	0	0	0	0	0
*O. nahuali*	1	2	2	0	0	1	1	0	2	1	0	0	1	1	0	1	0	1	1	0	1	1	2	0	1	0	2	1	1	1	2	0	0	2	1	0	2	0	1	0	0	1	1	1	1	2	-	0	0	0	0	0	0
*O. cavei*	1	1	2	0	0	0	0	0	2	0	0	0	1	0	0	2	0	2	1	0	1	1	2	0	1	1	2	1	1	0	1	0	0	1	1	0	2	0	1	1	2	0	0	1	1	2	-	?	?	?	?	0	1
*O. ratcliffei*	1	1	2	0	0	1	1	0	2	1	0	0	1	1	0	2	0	1	0	0	1	1	2	0	0	0	2	1	1	1	1	0	0	2	0	0	2	0	1	1	3	0	1	1	1	2	-	?	?	?	?	0	0
*O. toriyamai*	1	1	2	0	0	1	1	0	2	1	0	0	1	1	1	1	0	1	0	0	1	1	2	0	1	0	2	1	1	1	1	0	0	2	1	0	2	0	1	1	3	1	1	1	1	2	-	1	0	0	0	0	0
*O. mutenroshii*	1	1	2	0	0	1	1	0	2	1	0	0	1	0	1	2	1	1	0	0	1	1	2	0	1	1	2	1	1	1	1	0	0	2	1	0	0	0	1	1	3	0	1	1	1	2	-	?	?	?	?	0	0

Alar reduction is widely present in several unrelated genera and species of Passalidae. Brachypterism, together with the associated morphological modifications, shared by all species of *Ogyges*, is a potential synapomorphy of this taxon. Nevertheless, in order to clarify the relationships with *Proculejus*, which is a primarily brachypterous genus, we selected only one character, the humeral callus of elytra (character 43), to distinguish brachypterous species from the flighted outgroup species *Oileus sargi* and *Proculejus nudicostis*. The bluish iridescence (character 42), when present, may appear on various areas of the body; to avoid overweighting this character, we considered it only once in the analysis.

### List of selected characters

**Figure 1.**
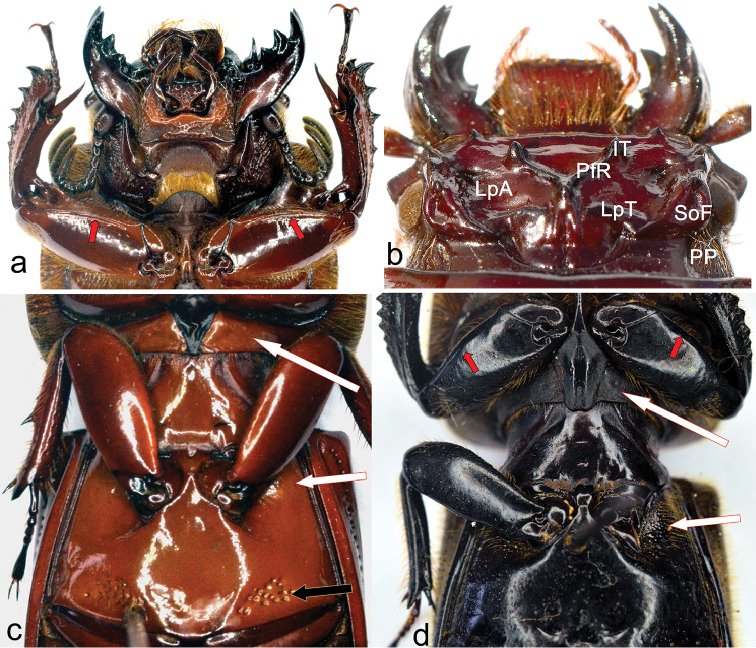
**a** *Ogyges crassulus*, ventral view. The anterior profemoral groove indicated with red arrows **b** *O. championi*, dorsal view. IT= internal tubercles; PfR= posterofrontal ridges; LpA= lateroposterior areas; LpT= lateroposterior tubercles; SoF= supraocular fossa; PP= postorbital pits **c** Sternum of *O. crassulus*. The white arrow indicates the smooth prepimeron and the bare anterior corners of metasternum; the black arrow indicates the patch of strong punctations posterior to the metasternal disc **d** Sternum of *Proculejus brevis*. The red arrows are signaling the absence of anterior profemoral groove. The white arrows indicate the chagreened (“greasy”) prepimeron and the slightly setose anterior corners of metasternum.

**Figure 2.**
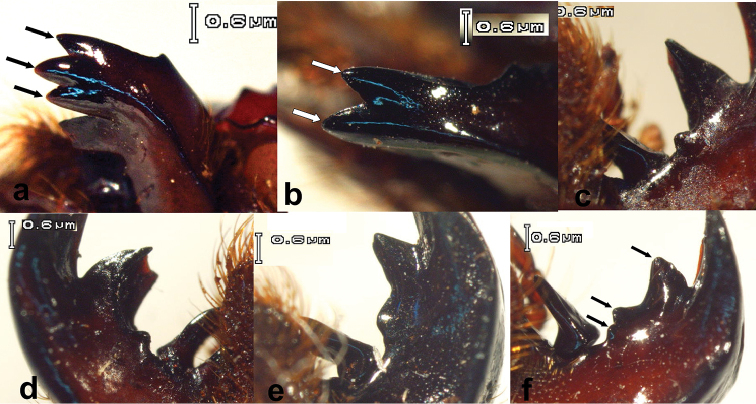
Mandibular teeth of Passalidae. **a** Tridentate mandibular apex of *Oileus sargi* **b** Bidentate mandibular apex of *Ogyges championi* **c** Right suprainternal tooth of *O. sargi* **d** Left suprainternal tooth of *O. sargi* **e** Right suprainternal tooth of *Proculejus sartorii* **f** Right suprainternal tooth of *O. championi*.

0. Frontoclypeal suture: (0) clearly present; (1) absent.

1. Clypeus: (0) delimitated from frons by a complete strong, transverse impression; (1) with a shallow and incomplete or insinuated delimitation with some granulations; (2) flat, without any indication of separation (although an abrupt change in plane is present in species with a vertical clypeus).

2. Clypeus: (0) very thick, forming a transversal and convex tumosity; (1) thin, tapering as a razor blade towards the apex; (2) same thickness all along, not thinned or thickened at apex.

3. Clypeus: (0) inclined; (1) vertical.

4. Small punctures (0.07 mm diameter) on frons: (0) absent; (1) present.

5. Internal tubercles: (0) present (Figure 1 b); (1) absent. The hyperthelic (over-developed) internal tubercles in *O. furcillatus* are fused to the, also hyperthelic, posterofrontal ridges (character 8).

6. Lateroposterior tubercles (Figure 1b): (0) keeled and well-marked; (1) with erased keel, barely marked.

7. Lateropostfrontal areas (also called frontal fossae) (Figure 1b) with granulations: (0) absent; (1) present, scarce (almost smooth); (2) present, densely abundant. When present, granulations are distributed on areas of the frons and/or vertex, particularly around the epicranial sutures (Boucher 2006). We assume covariation in this character and, in order to avoid double weight we only considered granulations on the frons, and not the clearly different surface texture near the epicranial sutures (character 10).

8. Posterofrontal ridges (Figure 1b): (0) absent; (1) present; origin (angle formed by the junction of posterofrontal ridges), not surpassing the eyes (*championi* type; Figure 4a); (2) present, defined or diffused; origin (“angle”) at level or surpassing the eyes (*laevissimus* type; Figure 4b). Although in the original description Schuster and Reyes-Castillo (1990) indicate that *O. furcillatus* lacks posterofrontal ridges, the assumption of presence of internal tubercles by the authors suggest a fusion of both characters. We assume the presence (as hyperthelic) in *O. furcillatus*. In *O. quichensis*, the development of the posteriorly massive center horn, at the level where the junction (“angle”) of the lateroposterior tubercles should be, obscures the presence of the character; nevertheless, most specimens have a ridge very posterior to the level of the eyes and we consider that, although not linear (due to a modification in the center horn, as in *O. furcillatus*) to be the posterofrontal ridge, corresponding to the *championi* type. In *P. pubicostis*, *P. nudicostis* and *O. cavei* the ridge is anterior (*laevissimus* type).

9. Posterofrontal ridges: (0) linear, clearly marked; (1) tumid on each side of the center horn, and then forming a clear (or diffuse) keel running to the sides of frons, marking the anterior margin of the lateropostfrontal areas (frontal fossae). State (1) has not been considered by Cano (2014) and Schuster and Reyes-Castillo (1990) as presence of the posterofrontal ridges (“quillas frontales” or frontal ridges). After a careful examination of tenerals and specimens cleared with KOH we conclude that state (1) of this character is homologous but distinct from character state (0) of the typical *O. championi*.

10. Area between laterofrontal tubercles and epicranial suture: (0) not shagreened; (1) shagreened.

11. Dorsal groove of center horn: (0) absent or indistinct (Figures 4b, 5a); (1) present, clearly marked (Figures 1b, 4a).

12. Length of center horn (base to tip): (0) short, not surpassing the level of eyes; (1) long, surpassing the level of eyes.

13. Sides of postfrontal groove: (0) shallow, at the same depth as lateropostfrontal areas; (1) deep, more than depth than the lateropostfrontal areas.

14. Supraocular fossae: (0) absent or, at most, a barely indicated impression, less than the length of half of an eye (Figure 5a); (1) present and longitudinal, forming two ridges, the internal wider than the external (Figure 5b); (3) present and widened posteriorly forming two ridges of approximately the same width (Figure 1b).

15. Postorbital pits (Figure 1b): (0) longitudinal and clearly marked, located just behind the eyes; (2) rounded, strongly marked, located just behind the supraorbital ridges, punctate-setose behind the eyes; (3) indistinct, punctate-setose behind the eyes.

16. Apex of ocular canthus: (0) acute; (1) rounded.

17. Antennal club (including all lamellae): (0) almost as long as wide; (1) wider than long, width of last antennomere at most 2.5 times its maximum length; (2) very wide, width of last antennomere at least three times its maximum length.

18. Antennal club (including all lamellae) in dorsal view: (0) flat; (1) concave.

19. Dorsal mandibular area facing dorsal tooth: (0) smooth; (1) granular; (2) granular punctate-striate.

20. Number of apical mandibular teeth: (0) three, same size (Figure 2a); (1) two, almost same size (Figure 2b).

21. Suprainternal teeth: (0) asymmetrical (Figure 2c, d); (1) symmetrical.

22. Left suprainternal teeth: (0) superior tooth large and bifurcate, with distant, extra-basal, very small, denticle (Figure 2d); (1) superior tooth large, connected to one small inferior tooth (Figure 2e); (2) superior tooth large, connected to one small bifid tooth (Figure 2f). Dentition of *Ogyges* and *Proculejus* seems to be very specialized and different from other Passalidae (with the probable exception of *Passipassalus*, an unrelated South American genus). The divided inferior portion of suprainternal teeth is here interpreted as an evolutionary novelty in *Ogyges* evolved from a common ancestor with *Proculejus*.

23. Medial basal mentum: (0) glabrous; (1) punctate-setose.

24. Lateral fossae of mentum: (0) shiny; (1) opaque.

25. Anterior ventral carina of ligula: (0) absent, central area tumid (arrow in Figure 4d); (1) present, complete, forming a plate (Figure 4c).

26. Pronotal shape: (0) quadrate; (1) rectangular, transverse, almost flat in dorsal view; (2) rounded, almost rectangular and very convex, with posterior sides angulate.

27. Lateral margin of pronotum: (0) with strong punctations (Figure 4 e); (1) impunctate (Figure 4f).

28. Lateral fossae of pronotum: (0) punctate-setose; (1) glabrous.

29. Rugose micropunctations (at a minimum magnification of 16x) on external border of lateral fossae of pronotum: (0) absent (Figure 4e); (1) present (Figure 4f).

30. Prosternelum: (0) shiny (only a small apical portion shagreened); (1) shiny medially (shagreened laterally); (2) opaque (completely shagreened).

31. Pre-epimeron (posterior procoxal bridge): (0) smooth (Figure 1c); (1) shagreened, appearing almost greasy (Figure 1d).

32. Mesosternal lateral scar: (0) longitudinal; (1) circular, apical; (2) absent.

33. Metasternal setation: (0) with abundant long setae running from mesocoxal cavities to posterolateral corner (Figure 5c); (1) with long setae only on anterior corners, but if reaching the marginal groove a glabrous patch partially separates the setation of anterior corners from that of the marginal groove (Figure 1d); (2) glabrous (Figure 1c). Some teneral specimens of *Ogyges* with a glabrous metasternum (e.g. *O. championi*, *O. marilucasae*, *O. quichensis*, *O. tzutuhili*, *O. nahuali* and *O. laurae*) have 1–2 minute, scattered setae.

34. Metasternal disc posteriorly: (0) smooth; (1) with patch of strong punctations without setae (Figure 1c); (2) with patch of small, setose-punctations.

35. Anterior profemoral groove: (0) present (Figure 1a); (1) absent (Figure 1d).

36. Metafemur: (0) elongate, at least three times as long as wide; (1) widened, at most 2.4 times as long as wide; (2) intermediate, between 2.5–2.8 as long as wide.

37. Posterior border of metatrochanter: (0) not grooved; (1) with small longitudinal groove (Figure 4h).

38. Posterior border of metatrochanter: (0) glabrous (1) with row of setae (Figure 4g).

39. Elytral dorsal striae: (0) all shallow, evident (Figure 3a); (1) all deep, evident, strongly punctate (Figure 3b); (2) elytral striae 1, or 1 and 2 deep, the rest barely visible or erased (Figure 3c).

40. Dorsal elytral punctures on striae 4 or 5: (0) visible at moderate magnification (16×), between 0.19–0.23 mm diameter, striae marked, punctures apparently connected by the unpunctured section of the striae; (1) visible only at high magnification (320×), between 0.15–0.19 mm diameter, striae unmarked or superficial; (2) minute (0.08 mm diameter), almost indistinct, visible only at high magnification (320×), striae well-marked; (3) Clearly visible or almost visible at naked eye or at low magnification (6.4×), between 0.3–1.0 mm diameter, area between punctures clearly connected with interestriae, striae well-marked.

41. Elytral surface: (0) shiny; (1) opaque.

42. Bluish surface reflections: (0) absent; (1) present.

43. Humeri between intervals 7–9: (0) without a distinct tumosity (Figure 5d), anterior half of elytra parallel; (1) with tumosity notably expanded laterally (“humeral callus”) (Figure 3), anterior half of elytra not parallel.

**Figure 3.**
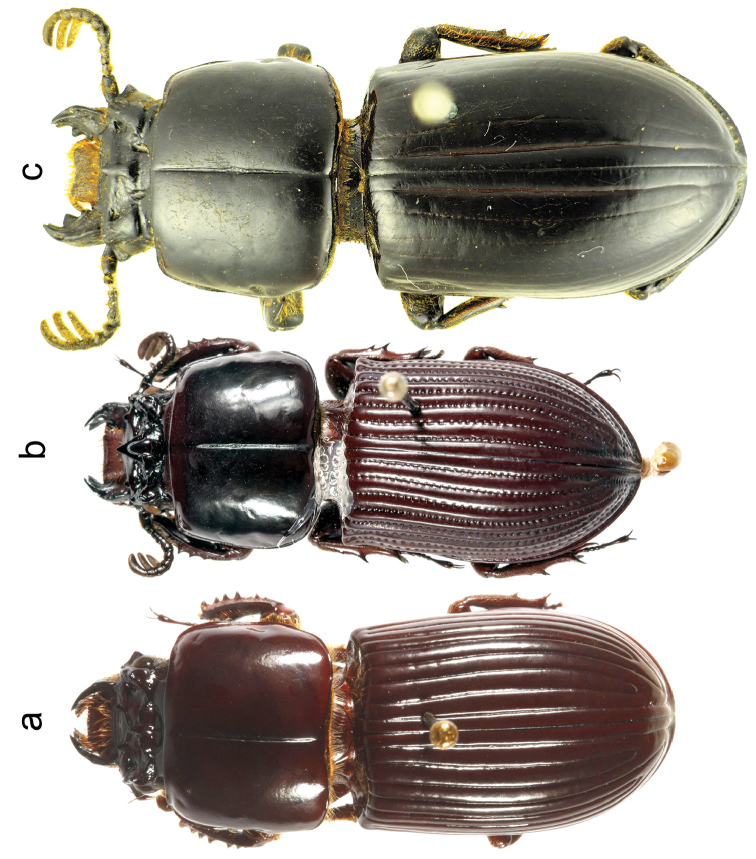
Dorsal habitus. **a** *Ogyges championi* **b** *O. monzoni*. **c**. *O. laevissimus*.

**Figure 4.**
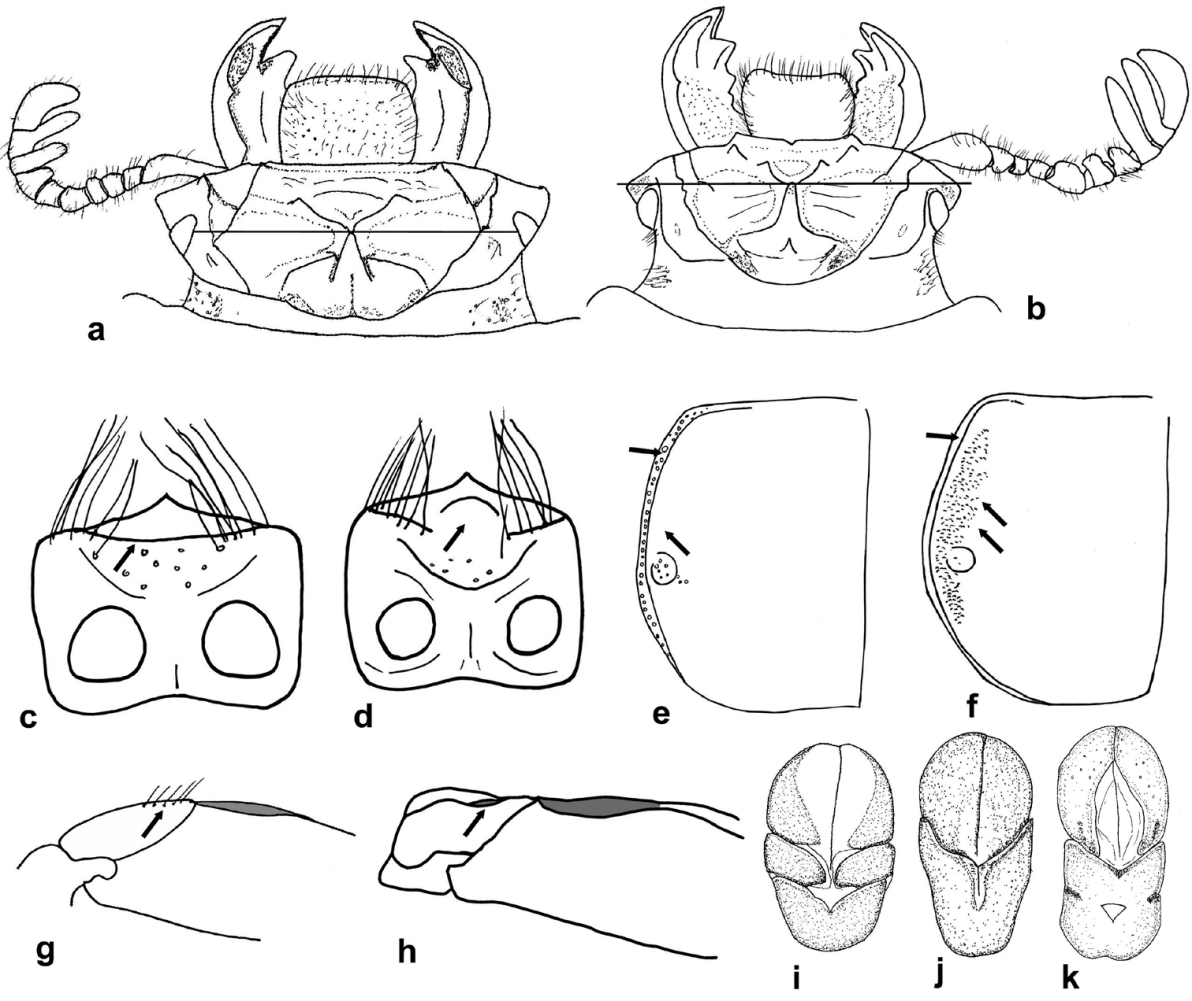
Passalidae, morphological details. **a** *Ogyges coxchicopi*, head. Transversal line indicates the origin (“angle”) of posterofrontal ridges **b** *O. sandinoi*, head. Transversal line indicates the origin (“angle”) of posterofrontal ridges **c** Anterior ventral carina of ligula of *O. championi* **d** Anterior ventral carina of ligula of *O. crassulus* **e** Laterodorsal view of pronotum of *Proculejus nudicostis* **f** Laterodorsal view of pronotum of *O. championi* **g** Posterior border of metatrochanter of *O. crassulus* **h** Posterior border of metatrochanter of *P. sartorii* **i** Ventral view of aedeagus of *O. monzoni* **j** Ventral view of aedeagus of *O. crassulus* **k** Ventral view of aedeagus of *O. laevissimus*.

44. Humeral setation (of the humeral callus): (0) setose; (1) glabrous.

45. Sides of elytra: (0) glabrous; (1) setose; (2) secondarily glabrous, with micropunctations of 0.08 mm. The presence of micropunctations (visible only at great magnification, 320× in teneral specimens) on glabrous elytra of *Ogyges* and *Oileus sargi*, suggest a secondary loss of setae in these taxa.

46. Sides of elytra: (0) setose on intervals 7–10 and all intervals on posterior declivity; (1) setose only on intervals 8–10.

47. Parameres and phallobase: (0) separated (Figure 4i); (1) fused or separation barely indicated only laterally (Figure 4j, k).

**Figure 5.**
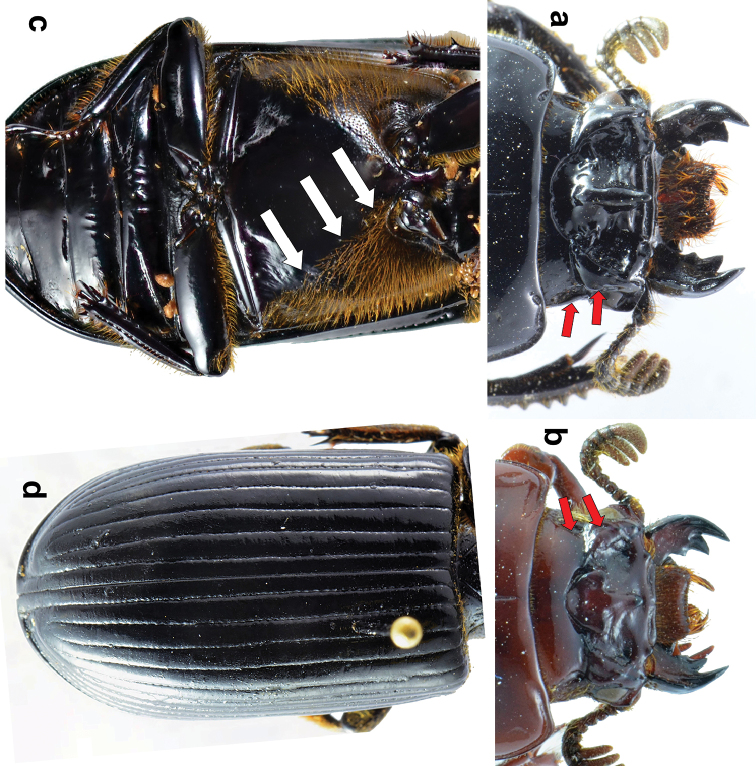
**a** *Proculejus pubicostis*, head. Arrows indicates the position of postorbital longitudinal pits and the supraocular fossae **b** *Ogyges laurae*, head. Arrows indicate position of the postorbital circular pits and the supraocular fossae **c** *Oileus sargi*, metasternum. Arrows indicate the distribution of metasternal setae **d** *Oileus sargi*, elytra.

48. Parameres: (0) separated medially (Figures 4i, j); (1) fused medially (Figure 4k).

49. Median lobe ventrally: (0) globose; (1) elongate.

50. Median lobe apicoventrally: (0) glabrous; (1) with minute setae.

51. Median lobe ventrally: (0) with longitudinal membrane; (1) sclerotized.

52. Total length of body: (0) medium-sized (26–34.5 mm); (1) large (35–46 mm); (2) small (18.71–25.5 mm). As measures of body size are variable, we treated the total length as a discrete variable (small/medium/large), based on average body length (error bars) of at least three specimens (one or two in species only known from these number of specimens).

The cladograms were constructed using TNT software (Goloboff et al. 2008). A preliminary analysis was conducted assigning all characters equal weights. We then tested the effect of homoplasy on the results by conducting different implied weights analyses (Goloboff 1993), with the constant of concavity (k) set to integer values from 1–12, where 1 was weighted most severely against homoplastic characters. Implied weights analyses were conducted using the heuristic “traditional search” algorithm of TNT, with 1000 replications and tree-bisection-reconnection branch-swapping (TBR), holding 1000 trees during each replication.

### Biogeographical analysis

The distribution of individuals of all species of *Ogyges* were plotted on a map, using ArcGIS 9.2. After the phylogenetic analysis, the range of each well-supported clade (but not of individual species) was colored. Barriers were hypothesized in relation to the dry (to moist) lowland valleys (principally below 1000 m in elevation) and major fault systems separating mountainous/volcanic ranges, and were analyzed and defined. The distributions of the individual species have been previously mapped by Schuster and Reyes-Castillo (1990: 15, 24, 30, 40), Schuster et al. (2005: 117), and Cano (2014: 25).

## Results

### Phylogenetic analysis

**Figure 6.**
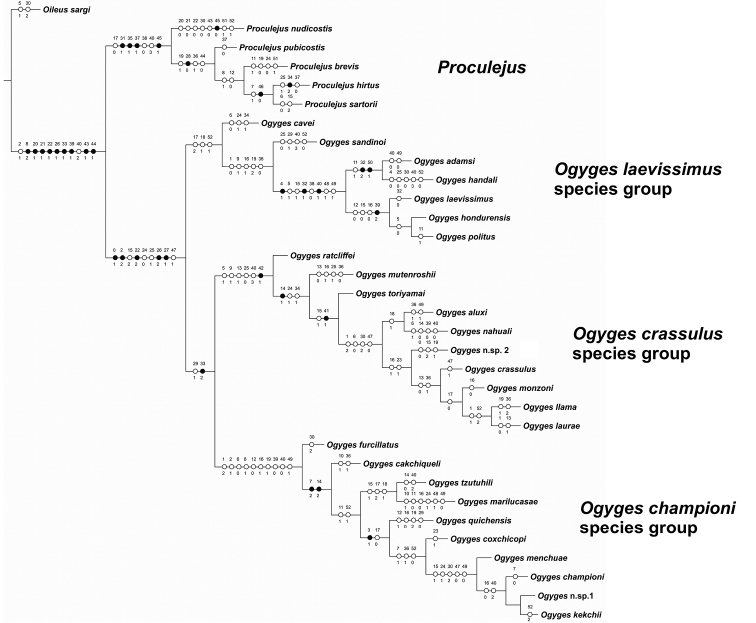
Cladogram of *Ogyges* obtained with concavity K=12, with character state changes indicated. The tree major clades were named as species groups. The 53 characters are named from 0–52. Black circles represent synapomorphies or autapomorphies; white circles represent homoplasies.

The analysis of the data matrix (Table 1) under equal weights led to six cladograms, with the constant of concavity (k) set at 3 led to three cladograms, and with k = 12 led to a single cladogram with 181 steps, CI of 0.403 and RI of 0.754 (Figure 6). In all the analyses, the 27 species of *Ogyges* were recovered as a monophyletic group, as generally occurred with the five species of the outgroup *Proculejus*. We recognize three main clades within *Ogyges*, named *O. laevissimus* species group, *O. championi* species group, and *O. crassulus* species group (Figure 6).

The synapomorphies that support the monophyly of *Ogyges* are the frontoclypeal suture absent [character 0(1); with a reversal in *Ogyges* sp. n. 2], the left suprainternal mandibular teeth with the large tooth connected to a smaller tooth divided in two [character 22(2)], the pronotum of rectangular and very convex shape [character 26(2)] and the lateral margin of pronotum without strong punctures [character 27(1)].

The *O. laevissimus* species group, with seven species, is supported by three non-synapomorphic character states: antennal club very wide [character 17(2)] and concave in dorsal view [character 18(1)]; and body large (35–46 mm) [character 52(1)]. These three states were resolved as parallelisms in the subclade *O. tzutuhili* + *O. marilucasae* of the *O. championi* species group. This species group is the sister taxon to the two remaining groups within *Ogyges*.

The clade containing the *O. crassulus* and *O. championi* species groups is supported by only two character states, presence of rugose micropunctuations (at moderate magnification) on external border of lateral fossae of pronotum [character 29(1)], with a reversal (absence) in *O. quichensis* and a parallelism (presence) in *O. sandinoi*; and the metasternum glabrous [probably secondarily glabrous; character 33(2)].

The *O. crassulus* species group, with ten species, is supported by six character states: internal tubercles absent [character 5(1)]; posterofrontal ridges tumid anteriorly at sides of center horn, forming a ridge extending towards the anterior margin of the frontal fossae [character 9(1)] with a parallelism in the *O. laevissimus* species group; sides of postfrontal groove deep [character 13(1)] with a reversal in *O. mutenroshii* and the clade of *O. crassulus* + *O. monzoni* + *O. llama* + *O. laurae*; anterior ventral carina of ligula with central area tumid [character 25(0)] with two parallelisms in the *O. laevissimus* species group; dorsal elytral punctures on striae 4 or 5 between 0.3–1.0 mm diameter and visible by naked eye [character 40(3)]; and bluish reflections present in all species [character 42(1)]. The characteristic patch of strong punctations on metasternum [character 34(1)] is absent in the basal species *O. ratcliffei*, but is a convergence in *O. cavei*. The wide punctures of the elytra [character 40(3)] apparently are a convergence (or symplesiomorphy?) with the species of the genus *Proculejus*.

The *O. championi* species group, with 10 species, is supported by ten non-synapomorphic character states: clypeus flat [character 1(2)] and thin [character 2(1)], lateroposterior tubercles keeled and well-marked [character 6(0)], posterofrontral ridges present and posterior in position [character 8(1)], center horn short [character 12(0)], apex of ocular canthus rounded [character 16(1), a reversal in *O. marilucasae* and the subclade *O. championi* + *O. kekchii* + *Ogyges* sp. n. 1], internal face of mandible granular [character 19(1)], dorsal elytral striae shallow and evident [character 39(0)], striae 4 or 5 with puntures of 0.19-023 mm diameter [character 40(0)], and the median lobe of aedeagus elongate [character 49(1)] (a reversal [49(0)] in *O. marilucasae* and the subclade *O. menchuae* + *O. championi* + *Ogyges* sp. n.1 + *O. kekchii*). The *O. championi* species group shares with some species of *Proculejus* the distinctive form of the mediofrontal structure (*sensu* Reyes-Castillo 1970), [character state 8(1)] and the short center horn [character 12(0)].

### Biogeographical analysis

**Figure 7.**
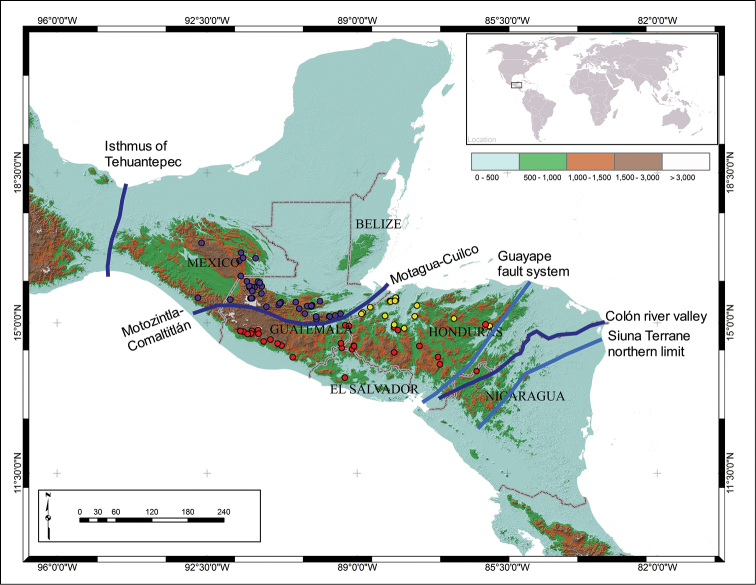
Distribution of the three clades of *Ogyges* in Nuclear Central America. Purple circles = *O. championi* species group; red circles = *O. laevissimus* species group; yellow circles = *O. crassulus* species group. Major barriers indicated with blue. Minor, or inconclusive barriers indicated with light blue.

Based on the cladogram (Figure 6) and the ranges of the species, *Ogyges* and its three consistent clades show clear distributional patterns (Figure 7). *Ogyges* is separated from the species of *Proculejus* by the dry valley of the Isthmus of Tehuantepec. The *O. championi* species group (Figure 7), with 10 species, is distributed in Chiapas, Mexico, in the northern mountain system from San Cristóbal de las Casas to Lagunas de Montebello, and in the southern system of mountains in the “El Triunfo” Biosphere Reserve; and in Guatemala, in the Sierra de los Cuchumatanes, Montaña Cuilco, Sierra de las Minas and Sierra de Santa Cruz. The distribution of this species group corresponds to the Maya block (Dengo 1969) whereas the other two species groups are endemic to the Chortis block (Dengo 1969). Apparently dryness (now and in past geological times) of the Motagua-Cuilco and Motozintla-Comaltitlán suture zones is the barrier separating it from the *O. crassulus* and *O. laevissimus* species groups, and corresponds (partially) to the subhumid corridor delineated by Stuart (1954).

The distribution of the *O. laevissimus* species group (Figure 7) extends mostly along the Pacific Volcanic Chain from Guatemala to El Salvador, to the north in Guatemala (Zacapa Department), and then to southeastern Honduras and northern Nicaragua, where the distribution corresponds approximately to the Southern Cordillera of the Honduran Chortis highlands as defined by Weyl (1980: 93–94) and highlighted by Townsend (2014: 214). This species group is separated from the majority of species of the *O. crassulus* species group by a series of intricate lowland (about 300–700m elevation) dry forests between the Central and Southern Cordilleras, perhaps related to the Ulúa-Chamelecón-Olancho system. However, two species of the *O. laevissimus* species group [*O. cavei* at Sierra de Agalta (Cerro La Picucha), and *O. adamsi* at Montaña Santa Bárbara] are sympatric with species of the *O. crassulus* species group, making the limits unclear. The eastern limit to the distribution of the *O. laevissimus* species group, and also of the genus *Ogyges*, appears to be the lowland moist to dry valleys of the Colón river in Nicaragua (0–700m) and its tributary, the Comalí river in Honduras (730–950m), or possibly the northern suture of the Siuna Terrane (Venable 1994) in Nicaragua (Figure 7). Alternatively, the moist (Atlantic) to dry (Central and Pacific) lowland (0–800m) Guayape fault system (Finch and Ritchie 1991) could be considered as a major barrier (Figure 7).

The *O. crassulus* species group, with 10 species, is distributed almost exclusively in northern Honduras, slightly extending to Guatemala at the Sierra del Merendón (Figure 7). It corresponds well with the Northern and Central Cordilleras of the Honduran Chortis highlands (Weyl 1980: 92–94, Townsend 2014: 214). The Guayape fault system (Figure 7) represents the eastern distributional limit of this species group.

## Discussion

### Phylogeny

The high homoplasy levels (CI=0.403) could be explained by the covariation of characters associated with flightlessness in taxa of Passalidae (reduced eyes, very narrow wings, and oval and fused elytra), but also because they have similar ecological niches (interior of rotten logs in humid forests). Flightlessness appears to have evolved several times in montane passalids, occurring in unrelated genera (e.g., *Passalus*, *Chondrocephalus*, *Veturius*, *Arrox*, etc.) and the body shape of passalids living in sapwood/heartwood tends to be convex (Johki and Kon 1987, Lobo and Castillo 1997, Kon et al. 2002).

In addition to the character used traditionally to separate *Ogyges* from *Proculejus*, the frontoclypeal suture, we consider the shape of the internal teeth of the mandibles, the punctate border of the pronotum, the sculpture of the prepimeron, and the lateral setation of the elytra to be the most relevant. Of these, until now, only the form of the internal teeth has proven to be stable and autapomorphic in *Ogyges* (also see Cano 2014). Nevertheless, a clearly marked frontoclypeal suture appeared only once in a terminal species (a reversal) of the *O. crassulus* species group, suggesting that the character is homoplastic in Passalidae.

The genus *Proculejus* urgently needs to be revised. At least two species, *P. nudicostis* Bates and *P. obesus* (Bates), do not share with the other species in the genus the bidentate mandibles, the laterally setose elytra and the shape of the internal teeth, characters traditionally used to diagnose the genus. Additionally, in one of our phylogenetic analyses (concavity k = 3, strict consensus), *P. nudicostis* was recovered as basal and excluded from *Proculejus*, bringing into question the monophyly of the genus.

Based on the phylogeny and distributions of more than twice as many species as were available to Schuster and Reyes-Castillo (1990: 40–45), we reject some of their groupings within *Ogyges* that were not based on phylogenetic analyses, and we suggest others that appear more natural and well-supported (Figure 6).

### Biogeography

*Ogyges* belongs to the Mesoamerican Montane cenocron (Morrone 2015). According to Halffter (1987), taxa belonging to it evolved in Nuclear Central America and then dispersed northwest and southeast from there. They have ancient South American affinities and are distributed mainly in montane cloud forests, although they penetrate occasionally into pine-oak forests. In the Oligocene-Miocene they dispersed from Central America northward (Halffter and Morrone 2017).

Regarding the vicariance between *Proculejus* and *Ogyges*, the Isthmus of Tehuantepec has been considered as a biogeographic break for several taxa (Marshall and Liebherr 2000, Morrone and Márquez 2001). A vicariant event during the Pliocene has been suggested as responsible for the divergence of several taxa, although an earlier vicariance at the end of the Miocene may have also occurred (Daza et al. 2010).

The Motagua-Polochic-Jocotán fault has been invoked as a sharp biogeographic break for vertebrate taxa (Castoe et al. 2009, Daza et al. 2010, Pérez-Consuegra and Vásquez-Domínguez 2015). We suspect that, for flightless passalids, although low elevation areas may be barriers, they are more effective when they are dry, at least at present. The Polochic suture zone valley (parallel to the north of the Motagua suture valley), is moister than the Motagua valley and does not separate species of passalids as well as does the Motagua; for example, three species of the *O. championi* species group are found on both sides of the Polochic suture zone (*O. tzutuhili*, *O. kekchii*, and *O. championi*). Here we recognize the Motagua-Cuilco (0–2000 m) system of dry valleys and the Motozintla-Comaltitlán suture zones (0–1900 m), as the major biogeographic barrier involved in the vicariance between the *O. championi* species group of the Maya block and the rest of the genus distributed in the Chortis block. The Motagua suture zone (although, together with the Polochic suture zone, according to authors) has been proposed as a barrier for several lowland and highland vertebrates from ~3–8 mybp (Daza et al. 2010: 351), or from ~4–5.5 mybp (Castoe et al. 2009: 95).

The distributional barriers between the *O. crassulus* and *O. laevissimus* species groups are unclear. Species of the *O. laevissimus* species group are distributed in the Quaternary Volcanic Chain of Guatemala and El Salvador, and the Tertiary Volcanic Southern Cordillera of the Chortis highlands in Honduras. But, again, the lowland dry valleys, such as the labyrinthic systems between the Ulúa and Chamelecón rivers and the Olancho Department in Central Honduras, merge as barriers. As to the timing of taxon divergence, Townsend (2014) suggests that most of the biota of Honduras could not have survived the mid-Miocene volcanic eruptions, when over 5,000 km^3^ of ignimbrites up to 2,000 m thick were deposited on top of the low-relief surface of the southern and western Chortis block, and tens of thousands of square kilometers were covered repeatedly in thick layers of ash (Townsend 2014). Thus, we assume that Honduran species of *Ogyges* would have to have originated after this event (~11–16 mybp).

The southern limit of distribution of *Ogyges* falls in the Sierra of Dipilto and Jalapa, Department of Nueva Segovia, in Northwestern Nicaragua, where mountains exceed 1500 m elevation. We (EBC, JCS) have collected passalids extensively in the cloud forests further south in the mountains (between 1200–1500 m) surrounding Jinotega and Matagalpa (Selva Negra, El Quetzal, Peñas Blancas, La Dalia and Datanlí-El Diablo) and in Granada at Mombacho volcano (1300 m), without finding a trace of *Ogyges*.

We suspect that future detailed studies of other taxa will confirm the vicariance hypothesis suggested by *Ogyges* in Nuclear Central America. Taxa with similar distributions include *Proculus* (Passalidae; Schuster et al. 2003), *Xylopassaloides* (Passalidae; Reyes-Castillo et al. 1987, Schuster 1993), Y*aaxkumukia* (Scarabaeidae; Micó et al. 2006), and the *integripennis* species group of *Geocharidius* (Carabidae; Sokolov and Kavanaugh 2014).

## Appendix 1

**Label data**

This section contains label data (verbatim) of specimens examined to obtain the character states. In brackets we completed some fragmented data.

***Oileus sargi* (Kaup)** (6 specimens). MEXICO: Chiapas, 30 mi N of Huixtla, #PST-1, 17 IV 1983, J.C. Schuster (1, UVGC). GUATEMALA: Chiquimula, Plan de la Arada, 1600m, 19 VII 1999, José Monzón, col. (1, UVGC); Escuintla, Fca. El Rosario, Volcán de Agua, 13–17 IX 1998, Alt. 1720m, #VNh-3, J.C. Schuster (1, UVGC); Quetzaltenango, Colomba, Costa Cuca, Volcán Lacandón, aldea Santa Anita 14°47'26"N, 91°43'30.6"W, 16 VI 2005, Cafetal, 1544m, E.B. Cano (1, UVGC); Zacapa, La Unión, 4-IV-1988, V. Gaitan (1, UVGC). HONDURAS: Ocotepeque Dept., El Portillo, VI 1992, 1900m, J.C. Schuster (1, UVGC).

***Proculejus nudicostis* Bates** (3). MEXICO: Omiltemi, Guerrero, 26-VII-65, G y V Halffter (1, INECOL); idem but 11-IV-63, G. Halffter, A, Barrera, A. Martinez, leg. (1, INECOL); Guerrero, 5 miles southwest Filo de Caballo, July 17, 1984, Carroll, Schaffner, Fruidlander (1, UVGC).

***P. pubicostis* Bates** (1). MEXICO: km 50 Teotitlán – Huautla, Oaxaca, 9-XI-68, P. Reyes, M. Cabrera, col., Alt 2400, bosque nebular. En tronco podrido muy húmedo (1, INECOL).

***P. hirtus* (Truqui)** (1). MEXICO: H[idal]go, La Mojonera, 28.X.1992., leg J. Pál. (1, UVGC).

***P. sartorii* Kaup** (7). MEXICO: Hidalgo, Tlanchinol, a 4 km del pueblo, bosque nuboso, 28 VII 2010, S. Orellana (1, UVGC); Hgo., Hwy. 105, 2.4 mi N Tlanchinol, 5000´, 8 May 1983, C.W. and L. O´Brien and GB Marshall (5, UVGC); Hidalgo, Tlanchinol, 26 V 78, madera de *Quercus* (1, UVGC).

***P. brevis* (Truqui)** (3). MEXICO: Oaxaca, carretera Llano de las Flores – Cerro Pelón, km 129, 17°26'38"N, 96°31'11"W, 2855m, 20 IX 1998, E.N. Smith, CONENS 9938 (1, UVGC); 10.9 km NE Cerro Pelón, Oaxaca, 26-II-84, alt. 2170m, bosque pino-encino, P. Reyes col. (2, INECOL).

***Ogyges adamsi* Schuster and Reyes-Castillo** (14). HONDURAS: Santa Bárbara, Montaña Santa Bárbara, VII.9.1968, 1968-12. Col. P. Adams (1 holotype, 1 paratype, INECOL); Teguc[igalpa], 28 XII 1980, in rotting tree, J.V. Mankins (1 paratype, INECOL); idem but 22-12-1980, in rotting trees, J.V. Mankins (2 paratypes, UVGC); 6-10-77, L. Yojoa, Hond., J.V. Mankins Collector, dibujo E. Aranda (1 paratype, INECOL); Francisco Morazán, arriba de El Rosario, Fca. La Tigra, 1700m, 20 VII 1991, J.C. Schuster, bosque nuboso, en tronco podrido (2 males, 2 females, UVGC); Rosario, San Juancito Mts., Honduras, Elev. 5120 feet, VII,[space],1930, Honduras exped, *O. yohoaensis* [unpublished name] (1 paratype, UVGC); III-2-1979, Mt. Ayuca, Honduras, Dept de F.M [Francisco Morazán], Coll. H.J. Marcus (2 paratypes, UVGC); San Juancito 7 mi SW, Francisco Morazán, Honduras, 16-VI-67, Coll. R.W. McDiarmid (1 UVGC).

***Ogyges aluxi* Schuster, Cano and Boucher** (26). HONDURAS: Cortés, Dept. + 30km W of Sn Pedro Sula, 1550m, 20–21 III 1987, J.C. Schuster (2, UVGC+ 11 UVGC paratypes); Cortés, N San Pedro Sula, 1650m, 2 X 2011, F. Camposeco, 15.586585N, -88.100232W (1, UVGC); Cortés, N of Cofradía, Cusuco, 1420–40m, 26 III 1991, J.C. Schuster (7 paratypes, UVGC); San Pedro Sula, Norte de Cofradía, P.N. El Cuzuco, 1900m, junio 1999, Monzón and Bailey (2, UVGC); Yoro, 10km, NO Morazán, 24 III 1991, 780m, J.C. Schuster (2, UVGC); Yoro, Sinaí, N15°26', W87°22', 1410m, Jun 2002, R.D. Cave (1, RC).

***Ogyges cakchiqueli* Schuster and Reyes-Castillo** (36). GUATEMALA: Huehuetenango, San Juan Ixcoy, 7 mi S de San Juan Ixcoy, 5 IV 1977, 2850m, J.C. Schuster col., bosque nebular de encinos #FF2 (1 paratype, INECOL); idem but S of San Juan Ixcoy, 8 VII 1977, 2280 m, J.C.S. col. (4 paratypes, INECOL); Huehuetenango, mountain above San Mateo Ixtatán, in log, 7 Feb. 1965, D.E. Breedlove, Collection of the CAS, San Francisco, Calif. (1 paratype, INECOL); Huehuetenango, San Mateo Ixtatán, 2975m, 2.6 miles of S. Mateo, 24 VII 1990, Brodie, Campbell (1, UVGC); Huehuetenango, Todos Santos, Villa Alicia, 2450m, 9–10 V 2002, A. Bailey (10, UVGC); Huehuetenango, Todos Santos, montaña sur de aldea Max, octubre 2001, bosque nuboso, J. Monzón (3, UVGC); Huehuetenango, Todos Santos, camino a Max, Rio Ocho, 2850m, 15.505477, -91.643826, J. Monzón, R. Anderson (2, UVGC); Huehuetenango, Santa Eulalia, 1kkm NW of Sta. Eulalia, 2750m, 15°45'49"N, 91°30'25"W, 2 III 1991, F.G. Thompson, S.P. Christman (1, UVGC); Huehuetenango, Fca. San Luis, 8km NE de Sta. Eulalia, 2490m, VII 1994, J.C. Schuster (pieces) (1, UVGC); Huehuetenango, Montaña de Cuilco, 2000m, bosque nuboso, 1996, M. Acevedo (4, UVGC); Huehuetenango, Cuilco Mt., above El Paraiso, 2100m, 9 III 1996, cloud forest, E.B. Cano (5, UVGC); Huehuetenango, Cuilco, arriba de Peña Roja, 2200m, 20 julio 1998, E.B. Cano, J. Monzón (3, UVGC).

***Ogyges cavei* Cano** (2). HONDURAS: Olancho, La Picucha, 11 km N Catacamas, 14.92740, -85.90983, bosque nuboso, 1800–2100 m, 8–14 V 2010. L. Sáenz (LSD 451) (holotype, UVGC); Comayagua, 10 km E Comayagua, 14.45973, -87.54609, Bosque nuboso, 2000 m, 15–19 V 2010. L. Sáenz (LSD 459) (paratype, UVGC).

***Ogyges championi* (Bates)** (153). GUATEMALA: Purula, Vera Paz, Champion, H.W. Bates, Biol. Cent. Amer. (1 lectotype, MNHN); Purula, Vera paz, B.C.A. 5.6. (1 paralectotype, BMNH); Baja Verapaz, Purulhá, 25 XII 1975, P. Reyes C. col. (4, INECOL); Idem but dibujo Aranda (1, INECOL); idem but 4-VI-93, alt. 1600m, bosque nebular, P. Reyes-Castillo, col. (3, INECOL); Baja Verapaz, Purulhá, Ranchitos del Quetzal, 2100m, 7 IX 2013, Centeno D. (1, UVGC); Baja Verapaz, Purulhá, Biotopo del Quetzal, Cerro Quisis, 9 IX 2001, bosque nuboso, 2000–2200m, tronco podrido, V. Ríos, R. Ávila y L. Benítez (4, UVGC; 2, USAC); idem but 16 IX 2001, 1700–1900m, L. Benítez (3, UVG); idem but 10 IX 2001 (3, UVGC; 1 USAC); idem but 16 IX 2001 (1, UVGC); Baja Verapaz, Purulhá, Biotopo del Quetzal, 19 IV 1998, E. Cano (7, UVGC), idem but 15 XI 1988 (1, USAC); idem but 26 V 1987 (1, USAC); idem but 15 XI 1988, M. Barrios (1, USAC); idem but 11 V 2000, A. Higueros (1, USAC); idem but agosto 2000, A. Higueros (1, USAC); idem but 1700m, 12 VI 2000, A. Higueros (1, USAC); idem but 6 V 2000 (1, USAC); Baja Verapaz, Purulhá, 9–10 III 1996, L. Rivera (1, UVGC); Baja Verapaz, Purulhá, nr. Biotopo, 1 XI 1989, J. Schuster (2, UVGC); Baja Verapaz, nr. Purulhá, 9 V 1976, 1640m, J. Schuster (2, UVGC); idem but 6 VII 1984 (3, UVGC); idem but 23 VI 1980 (1, UVGC); Baja Verapaz, Purulhá, VII 1984, J. Schuster (1, UVGC); idem but 5–6 IX 1992 (1, UVGC); Baja Verapaz, Purulhá, marzo 1996, F. Rivera (1, UVGC); Baja Verapaz, Purulhá, 1570m, 24 VI 1980, Col. J. Schuster (1, UVGC); Baja Verapaz, km 156, Purulhá, 13 IX 1981, C. Porter (1, UVGC); Baja Verapaz, Purulá, 5km E Purulhá, 1530–1650m, 22/24 julio 1977, E. Fisher, P. Sullivan (1, UVGC); Baja Verapaz, Purulhá, km 156–160, 12–13/III/1994, C. Cardona (2, UVGC); Baja Verapaz, 5 mi E Purulhá, 11 VII 1991 (6, UVGC); Baja Verapaz, Purulhá, VII 1984, J. Schuster (1, UVGC); idem but 1570m, 24 VI 1980 (1, UVGC); Baja Verapaz, Purulhá, km 163.5, 15 III 1986, A. López (1, UVGC); Baja Verapaz, Purulhá, 9–10 III 1996, E. Kepfer (4 , UVGC); Baja Verapaz, Purulhá, Biotopín, 1500m, junio-julio 1998, 15.799002E, 16838959N, C. Bailey, J. Monzón (6, UVGC); Baja Verapaz, 5 mi E. Purulhá, 11 VII 1991, P. Hubbell (2, UVGC); Baja Verapaz, Purulhá, Hotel del Biotopo [posada Montaña del Quetzal], 5 VIII 1988, R. Pérez (2, UVGC); idem but 8 VIII 1988, J. Schuster (1, UVGC); Baja Verapaz, Purulhá, Biotopo del Quetzal, 1–18 VII 1997 (1, UVGC); Baja Verapaz, Purulhá, 4 III 1988, R. Mora (1, UVGC); Baja Verapaz, Cerro Verde, 1700m, 16 VI 2000, A. Higueros (1, UVGC); idem but 13 VI 2000, 1900m (1, USAC); idem but 1800m (5, USAC); idem but 8 VI 2000, 1800m (1 USAC); idem but La Unión Barrios, Cerro Verde, 1800m, VII 2000, A. Higueros (1, UVGC); idem but VIII 2000 (1, UVGC); Baja Verapaz, Purulhá, Cerro Verde, 30 IX 2001, b. nuboso, 2000–2200m, tronco podrido, transecto 3, L. Benitez (1, UVGC); Baja Verapaz, Unión Barrios, Cerro Verde, 11 XI 1978, M. Dix (2, UVGC); Baja Verapaz, “Cobán”, Purulhá, 9 V 1991, M. Centeno (1, UVGC); Baja Verapaz, 4 km SW Purulhá, 1900m, 3 XII 1991, leg. R. Baranowski (1, UVGC); Baja Verapaz, 5 mi E Purulhá, upper tropical, 11 VII 1991, P. Hubbell (1, UVGC); Baja Verapaz, Chilascó, VI 1998, bosque nuboso (1, UVGC); idem but VI 1996 (1, UVGC); Baja Verapaz, 7km al S de Sta. Elena 3 XI 1979, M. Monzón (1, UVGC); idem but, F. Asturias (1, UVGC); Baja Verapaz, Unión Barrios, IX-1975, alt. 1440m, E.C. Welling col. (2, INECOL); Baja Verapaz, Finca las Nubes [Sierra de las Minas], 2400m, 22 VI 1993, M.C. Paiz (1, UVGC); Alta Verapaz, Tactic, a más de 1400m, VI-76, E.C. Wellig col. (7, INECOL); Alta Verapaz, Senahú, 1350m, Fca. El Volcán, 21 VI 1984, J. Schuster, hembra [15.504484, -89.866665](2, UVGC); Alta Verapaz, Senahú, Finca El Volcán, 25 III 1997, C. Estrada, E. Curley, R. Mejía (3, UVGC); idem but 12 XI 1984, C. MacVean, 1500m (2, UVGC); Alta Verapaz, 21 km San Cristobal Mexabaj, 1570msnm, 4 III 1989, J. Schuster (4, UVGC); idem but 3 III 1989 (2, UVGC); Alta Verapaz, Chelemhá, VI 1989, F. Herrera (2, UVGC); Alta Verapaz, Cobán, 10 XII 1993, Arturo Godoy (2, UVGC); Alta Verapaz, municipio de San Juan Chamelco, campamento Ecoquetzal, aprox. 1 hora en carro y 3 horas a pie de S.J. Chamelco, VI 1996, J.C. Schuster (2, UVGC); Alta Verapaz, 7–10/V/2010, A. Monterroso (1, UVGC); Alta Verapaz, Col. G. Kramer, 1976 (1, UVGC); Zacapa, Sierra de las Minas, 17 XI 1994, E. Curley (1, UVGC); idem but 1800m, 18 VII 1994 (2, UVGC); Zacapa, San Lorenzo, Sierra de las Minas, Cerro del Mono, 9 VI 1993, 2200m, J. Monzón (2, UVGC); Zacapa, Sierra de las Minas, 1800m, 20 III 1997, A. Pinelo (1, UVGC); Zacapa, San Lorenzo, VII 1986, D. Hernández (1, UVGC); idem but 10 XI 1986 (1, UVGC); Zacapa, San Lorenzo, 8 IV 1982, J. Schuster (2, UVGC); Zacapa, Sierra de las Minas, 2200m, III 2007, Col. R. Gramajo (1, UVGC); Zacapa, San Lorenzo, Sierra de las Minas, 17 IV 1994, X. Segovia (1, UVGC); Zacapa, Sierra de las Minas, 23 IV 1995, H. Pezzarosi (1, UVGC); Zacapa, Sierra de las Minas, San Lorenzo, 4 X 1987, bosque húmedo encino-pino, 1900m (1, UVGC); Zacapa, 12–19 III 2001, A. Monterroso (1, UVGC); Zacapa, Sierra de las Minas, zona núcleo, IV 2007, M.J. Larrave (1, UVGC); Zacapa, 4 mi N of San Lorenzo 18 IV 1981, 2110m, J. Schuster (1, UVGC); idem but 8 IV 1982, 2165m (1, UVGC); idem but 2150m (1, UVGC); El Progreso, Cerro Pinalón, arriba de los Albores, 5 V 1990 (1, UVGC).

***Ogyges coxchicopi* Schuster, Cano and Boucher** (18). GUATEMALA: Izabal, nr. Rio Zarco, above El Arenal, #EER-C, 11–20 IV 1993, Col. Enio Cano (holotype, UVGC); same data (13 paratypes, UVGC); Zacapa, rd. to Jones, Fca. Monte Morán, 8 IV 1983, S. Ubico, 1600m. alt, #PNO-5 (4, UVGC).

***Ogyges crassulus* (Casey)** (74). GUATEMALA: Izabal, Morales, above San Antonio, 4–7 VIII 1994, C. Guirola, E. Smith (7, UVGC); Izabal, Los Amates, Cerro Nylon, 8–11 IV 1990, J.C. Schuster #WIe-2, 1120m, alt. (11, UVGC); idem but 8-IV 1990 (1, UVGC); idem but 11 IV 1990 (2, UVGC); idem but 8–11 IV 1990, 1240m (1, UVGC); Izabal, Morales, Sierra de Caral, finca Firmeza, 450m [?], J. Monzón (1, UVGC); idem but Caral, 1150m, 7–14 VIII 1994 (1, UVG) ; idem but, Caral, Negro Norte, V 1995, J.Monzón, 1200m (2, UVGC); idem but 1200m, abril 1997 (6, UVGC); idem but, 27 Junio 1998, 1150m, E.B. Cano, J.Monzón (6, UVGC); idem but Sierra Caral, 1150m, 15°22.67N, 88°41.68W, 1150m, 30 VIII 1997, J. Monzón (13, UVGC); Idem but 25 III 1997, J.Monzón, A.C. Bailey (4, UVGC); Izabal, Morales, Negro Norte, Finca Firmeza, 27 VI 1998, 1150m, E.B. Cano (6, UVGC); Izabal, Morales, S de Caral, aldea Negro Norte, cerro del Aguacate, 23–27 VI 1991 (9, UVGC); Izabal, Morales, Sierra Caral, quebrada La Firmeza, 1–2 IV 1992, Col. C. Guirola (1, UVGC); Izabal, 30km SSE of Morales, 15°21.368'N, 88°40.726'W, 1240m, under wood, 21 July 2007, R.S.Zack Coll. (1, UVGC). HONDURAS: Cortés, Merendón, 15°30'12"N, 88°11'54"W, 7 June 2003, R.Turnbow (1, UVGC); Cortés, P.N. Cusuco, UTM 16P377265 1713975, ALT 1235M, 25–29 VI 2006, dung baited pitfall trap, col. K. Draysob (1, UVGC).

***Ogyges furcillatus* Schuster and Reyes-Castillo** (47). GUATEMALA: Zacapa, 5 mi N S. Lorenzo, 17 VI 1981, W. Dix (holotype, UVGC); Zacapa, San Lorenzo, 2100m alt. 18 IV 1981, J.Schuster, bosque nebular #NR-1, dibujo F. Aranda (1 paratype, INECOL); Zacapa, above San Lorenzo, 1–15 IV 1993, # EERB, Enio Cano, col. (1, INECOL); Zacapa, Sierra de las Minas, Fca. Santa Clara, Cerro Pinalón, 2500m, B. nuboso, 23–24 VIII 1998, E. Cano (2, UVGC); El Progreso, arriba Albores, Cerro Pinalón, 1–7 marzo 1993, Col. E. Cano, #EER89 (1, INECOL; 8, UVGC); idem but 24–27 Feb. 1993, #EER (7, INECOL; 6, UVGC); El Progreso, Cerro Pinalón, arriba de Los Albores, 25 II 1990, J. Monzón, C. Granizo (6, UVGC); idem but 28 III, 1992, #WN, J.C. Schuster (2, UVGC); idem but 2450m, 6–9 VIII 1991, without collector (2, UVGC); idem but 7 VII 1990, A. Joachim, trampa pitfall (2, UVGC); idem but 7 VIII 1990, I. Flores, T. pitfall (1, UVGC); Progreso, above Los Albores, Fca. Las Nubes, 2 VII 1993, 2500m, M.C. Paiz (6, UVGC); “Guatemala”, Aug 77, M. Mogollón, col., 11364 (1 paratype, INECOL).

***Ogyges handali* Cano** (49). GUATEMALA: Chiquimula, aldea Santa Rosalía, El Duraznal, cerca del Plan de la Arada, 11 VI 2011, 14°31'30.4'’N, 89°22'47.3'’W, 1668 m, bosque nuboso, Col. E.B. Cano (holotype, UVGC); same data (2 paratypes, UVGC); same data except 1–4 IV 2011, aprox. 1700 m, Col. C. Suchité (11 males, 14 females, paratypes, UVGC); same data except aldea El Duraznal, cerca de La Mesilla, 1630 m, 20 VIII 1998, Cols. E. Cano and J. Monzón (5 paratypes, UVGC); Chiquimula, San José las Minas, camino entre caserío Las Presas y el Plan de la Arada, Bosque nuboso. 1900 m, 24 VI 1998, Col. E. Cano (16 paratypes, UVGC).

***Ogyges hondurensis* Schuster and Reyes-Castillo** (9). HONDURAS: Dept. Ocotepeque, Montaña “El Portillo”, 13 IV 1988, J.Schuster, 1900m, bosque nebular, dibujo E. Aranda (holotype, INECOL); Ocotepeque Dept., El Portillo Mtn., 2 VII 1985, 1900m, J.C. Schuster (1 paratype, UVGC); idem but 13 IV 1981 (1 paratype, UVGC); idem but 19 VII 1981 (1 paratype, UVGC); idem but 27 VII 1981 (1 paratype, UVGC); idem but 13 IV 1981 (1, UVGC); Intibuca Dept., 7 mi E. La Esperanza, 1740m, 7 IV 1977, #L.V., J.C. Schuster, Col. (2 paratypes, INECOL); F. Feb 79, L. Volcán S.S., Consuelo J.M. (1 paratype, INECOL).

***Ogyges kekchii* Schuster and Reyes-Castillo** (38). GUATEMALA: Baja Verapaz, Purulhá, 13–24 VI 1980, J.C.S. #MI-1 male, cloud forest, 1570m alt. (holotype, INECOL); Baja Verapaz, nr. Purulhá, 27 X 1985, P. Mayorga (1 paratype, UVGC); Baja Verapaz, Purulhá (km 150), 23–24 VI 1980, J.C. Schuster, cloud forest, 1570m alt. (3 paratypes, UVGC); Baja Verapaz, Purulhá, Biotopo del Quetzal, 15 XI 1988, Col. M. Barrios (4, USAC), idem but 25 VII 1987, 2100m (2, USAC); idem but Agosto 2000, A. Higueros (2, USAC); idem but, Cerro Quisis, 2200m, 28 VII 2001, V. Ríos, R. Ávila y L. Benítez (5, USAC; 3 UVGC); idem but 2000–2200m, 30 IX 2001 (2, USAC; 2, UVGC); idem but 9 IX 2001 (2, USAC; 3 UVGC); Baja Verapaz, Purulhá, Posada del Quetzal 15 IV 1988, E. Cano (1, UVGC); Baja Verapaz, Purulhá, en tronco podrido 4 VI 1993, E. Padilla (1, UVGC); Baja Verapaz, Purulhá, Biotopo del Quetzal 1–18 VII 1997 (2, UVGC); Baja Verapaz, Guaxabajá, RBSM (Sierra de las Minas), junio 2002, 2640m, 15°08'42"N, 89°57'20"W, Col. A. Higueros (1, USAC); Alta Verapaz, Tactic, VI 1976, 1400m, E.C. Wellig col. (1 paratype, INECOL); Alta Verapaz, Mpio. Tucurú, Chelem Há, 2200–2300, Mt. Yalijux, bosque nebular, 7 V 1989, G. Ibarra (1, UVGC); idem but 22 III 1989 (1, UVGC); Alta Verapaz, Chelem Há, 22 III 1989, José Monzón (1, UVGC); Alta Verapaz, Senahú, aldea Secampana, Las puertas, montaña Yalijux, 15°24'34"N, 90°02'50"W, 23 V 1999, 1880m, S. Pérez (1, UVGC); Alta Verapaz, San Juan Chamelco, campamento Ecoquetzal, VI 1998, 2300m, J.C. Schuster (1, UVGC).

***Ogyges laevissimus* (Kaup)** (50). GUATEMALA: Volcán de Agua, Departamento Antigua, 8-VI-74, J. Hendrichs col., altitud 2600–3000m, caminando (10, INECOL); V[olcán] Atitlán, Depto. Sololá, 29 I 1977, C. MacVean col. 2500m (1, INECOL); Sololá, Nahualá, VIII-75, a más de 2350m alt., E.C. Welling col. (5, INECOL); Quetzaltenango, Cantel, VIII 1978, más de 2200m, E.C. Welling (1, INECOL); idem but VIII-1975 (12, INECOL); Quetzaltenango, VIII 1975, a más de 2200m, E.C. Welling (3, INECOL); idem but VI 1976, alt 2200m (1, INECOL); Quetzaltenango, Zunil, 2000m, VII 76, E.C. Welling (17, INECOL).

***Ogyges laurae* Cano** (11). HONDURAS: Olancho, 11 km N of Catamacas, mountain “La Picucha”, 14.92740, -85.90983, 1800–2100 m, 8–14 V 2010. L. Sáenz collector (LSD) (holotype, UVGC); idem (10 paratypes, UVGC).

***Ogyges llama* Cano** (44). HONDURAS: Cortés, cerca de San Pedro Sula, 15.512598°, -88.113660° 1580 m, 2 X 2011, bosque nuboso. Coll. F. Camposeco (holotype, UVGC); idem (42 paratypes, UVGC); Cortés Dept. 30 km W of S. Pedro Sula, 1550 m alt., 20–21 III 1987, bosque nuboso, J.C. Schuster, # VI3 female, Type C disturbance sound (1 paratype, UVGC).

***Ogyges marilucasae* Reyes-Castillo and Castillo** (27). MEXICO: Chiapas, El Triunfo, 12-V-85, M. Vertiz (2, IBUNAM); idem but 24-I-85, F. Arias (3 paratypes, IBUNAM); idem but 23-I-85, F. Arias (4 paratypes, IBUNAM); idem but Reserva El Triunfo, 9 julio 1993, S. Zaragoza (11, IBUNAM); idem but 24-I-85, M. Vertiz (1 paratype, IBUNAM); idem but 23-I-85, H. Velazco (1 paratype, IBUNAM); idem but 23-I-85, M. Vertiz (1 paratype, IBUNAM); Reserva “El Triunfo”, campamento “El Triunfo”, 23-VIII-96, B. Gómez y Gómez (3, INECOL); Mpio. Jaltenango, Reserva El Triunfo, 29 IV 1992, B. Gómez Col. (1 INECOL).

***Ogyges menchuae* Cano** (36). GUATEMALA: Quiché, Uspantán, aldea Laj Chimel, montaña al norte de la aldea, 2100 m., VII 1998, bosque nuboso. E.B. Cano (holotype, UVGC); idem (8 paratypes, UVGC); idem, except Norte Laj Chimel, San Pedro, 2100m (2 paratypes, UVGC); idem but aldea Laj Chimel, A.González-Madrid, 30 V 2011 (13 paratypes, UVGC); idem but, Aldea Laj Chimel, road to San Pablo, 30 IV 2011, 15°27'30.69'’N, 90°46'26.11'’W, 2035 m, A. Gónzález-Madrid 30 V 2011 (4 paratypes, UVGC); same data except, Aldea Laj Chimel, road to San Pablo, 30 IV 2011, 15°27'30.69'’N, 90°46'26.11'’W, 2035 m (6 paratypes, UVGC); same data except, Montaña El Amay, in logs 15 VI 2012, A. Zamora (2 paratypes, UVGC).

***Ogyges monzoni* Schuster, Cano and Boucher** (14). GUATEMALA: Izabal, Morales, above San Antonio, 4–7 VIII 1990, C. Guirola, E. Smith (2 specimenes, including holotype, UVGC); same data (10 paratypes, UVGC); Izabal, Los Amates, Cerro Nylon, above San Antonio, 1295m alt., 11 IV 1990, #WI-2, J.C. Schuster (1, UVGC); idem but, 1250m, 8–11 1990, J.C. Schuster (1). HONDURAS: Cortés, Parq. Nac. Cusuco, 5 km W Bs Aires, 26–27 agosto 1994, R.D. Cave (1).

***Ogyges mutenroshii* Cano** (2). HONDURAS: Cortés, Parque Nacional Cusuco, 2nd. broadleaved forest. nr. UTM 16P 369210 1713408. Ca. 1656 m alt., 07–11 VIII 2007. Coll. S. Beynon (holotype, UVGC); idem except UTM 16P 369795 1714017 alt. aprox. 1550 m. 16–20 VII 2006, dung baited pitfall trap, Coll. E. Marabuto (1 paratype, UVGC).

***Ogyges nahuali* Schuster, Cano and Boucher** (5). HONDURAS: Olancho, route La Unión – El Dictamo, 16 km La Muralla, 1550 m, VII 1995, T. Porion & A. Grange (holotype, MNHN); Olancho, Parque Nacional La Muralla, 17 IX 1995, R. Lehman (1 paratype, UVGC); Olancho, Parque Nacional la Muralla, Sendero Pizote, 10 VI 2003, R. Turnbow (1 paratype, UVGC); P.N. La Muralla, 15.07N, 86.45W, 16 Jul 1998, col R. Cave (1, RC); Olancho, La Picucha, 11 km N Catacamas, 14.92740, -85.90983, bosque nuboso, 1800–2100 m, 8–14 V 2010, L. Sáenz (1, UVGC).

***Ogyges politus* (Hincks)** (18). EL SALVADOR: H. Mte. Cristo, 2200m, Dr. A. Zilch, S. 1951 (Paratype, UVGC); Trifinio, 22-VII-70, Virkki, citogenética (1, INECOL); Idem but 27/7–60 (1, INECOL); idem but S. Ana, Trifinio, 8.III.1960. Réc.: J. Bechyné (2, one also with label: dibujo E. Aranda, INECOL); Hacienda Montecristo, Metapán, 13 mayo 1973 (1, UVGC); Chalatenango, 18-III-99 (1, UVGC); GUATEMALA: Chiquimula, camino entre San José Las Minas y al Plan de la Arada, 24 VI 1998, 1640–1910m (5, UVGC); Chiquimula, aldea Santa Rosalía, 14.49027N, -89.368611W, 1700m, 1-IV 2011, C. Suchité (4, UVGC); Santa Rosa, Pueblo Nuevo Viñas, Cerro Miramundo, finca Miramundo, 27–28 V 1999, E.B. Cano, bosque encino casi nuboso (algunos pinos) (3, UVGC).

***Ogyges quichensis* Schuster and Reyes-Castillo** (20). MEXICO: Chiapas, Municipio de Ocosingo, second ridge NE of Las Margaritas, above La Soledad, 1828m, 1 VII 1981, D.E. & P.N. Breedlove (1 paratype, INECOL). GUATEMALA: Quiché, 9 mi ruta CA 7 hacia Nebaj, 19-X-1975, M. Dix (holotype, UVGC); Quiché, Uspantán, aldea Laj Chimel, 4 V 2006, 2300m, J. Rivas (3, UVGC); idem but norte Laj Chimel, San Pedro 2100m, VIII 1998, E. Cano & J. Monzón (1 UVGC), idem but aldea Laguna Danta 1 VI 1998, E. Cano (5 UVGC), idem but 2200m, 15 septiembre 2008, Monzón & Anderson (3 UVGC); Huehuetenango Dept., 9 mi Oeste de Barillas, 7 IV 1977, alt. 2250m #FK, J. Schuster, col. (1 paratype, INECOL); Huehuetenango, Todos Santos, Villa Alicia, 2450m, 9–10 V 2002, Col. A. Bailey (2, UVGC); Huehuetenango, Nentón, Yalambojoch, Reserva Ixcansán, 15.68176N, -91.23022W, 1800m, 14 VI 2013, M.Acevedo (2, UVGC); Huehuetenango, Soloma, Cerro Cruz Maltín, aldea Crinolina, 1900m, 18 V 2002, J.Monzón (1, UVGC).

***Ogyges ratcliffei* Cano** (1). HONDURAS: Olancho, La Picucha, 11 km N Catacamas, 14.92740, -85.90983, bosque nuboso, 1800–2100 m, 8–14 V 2010, L. Sáenz (LSD 451) (holotype, UVGC).

***Ogyges sandinoi* Cano** (5). NICARAGUA: Nueva Segovia, nr Jalapa, Cerro Jesús, 13.98079, -86.17922, 28–31 May 2011, L. Sáenz collector (LSD) (holotype, UVGC); 4 paratypes with the same data (3, UVGC; 1, USAC).

***Ogyges toriyamai* Cano** (8). HONDURAS: Comayagua, Parque Nacional Cerro Azul Meambar, 14.87140, -87.90036, bosque nuboso, 800–1120 m, 20–24 V 2010, L. Sáenz. LSD 471 (holotype, UVGC); 2 paratypes with the same data, UVGC; Comayagua, Parque Azul Meambar, Agosto 30, 1996, Col. Marco Mendoza (1 paratype, UVGC); Santa Bárbara, Parque Nacional Santa Bárbara, 18 IV 2014, Yoshimoto leg (1, JYC); same data but 16 II 2014 (1, JYC); same data but buffer zone, 10 III 2013 (2, JYC).

***Ogyges tzutuhili* Schuster and Reyes-Castillo** (42). GUATEMALA: Alta Verapaz, Salamilá, 7 IV 1982, G. Ibarra (holotype, INECOL); Alta Verapaz, Tucurú, Chelemhá, 2200–2300m, Mt. Yalijux, B. nebular, G. Ibarra, 4 V 1989 (7, UVGC), Alta Verapaz, Chelemhá, marzo 1989, José Monzón (1, UVGC); idem but 22 VI 1989 (1, UVGC); idem but 22 III 1991 (1, UVGC); idem but 22 III 1989 (1, UVGC); Alta Verapaz, Secampana, Finca Esperanza, Q´anixul, 27 VI 1999, b. nuboso, S. Pérez (1, UVGC); Alta Verapaz, San Juan Chamelco, Campamento Ecoquetzal, aprox. 1 hora en carro y 3 horas a pie de S.J. Chamelco, VI 1998, J.C. Schuster (3, UVGC); Zacapa, San Lorenzo, 1–15 IV 1993, #EER-B, Enio Cano, col. (6, INECOL; 17 UVGC; 1 MNHN); Quiché, Uspantán, aldea Laj Chimel, 2000m, junio 1998, ex bosque nuboso, E.B. Cano (2, UVGC).

***Ogyges* sp. n. 1** (296). MEXICO: Lagunas de Montebello, Mpio. La Trinitaria, Edo. de Chiapas, 1-IX-81, 1556m, P.R.-Castillo et al. cols. bosque mesófilo (1 male, INECOL); idem but 1470m, C. Castillo, col. (1, INECOL); Chiapas, Lagunas de Montebello, 5 VIII 1991, C. Mayorga (1, IBUNAM); Chiapas, Santa Rosa, VIII-62, G. Halffter leg (115, INECOL); Edo. de Chiapas, Santa Rosa, 20 V 1967, G. y V. Halffter y P. Reyes Cols. (18, INECOL); idem but 14–22 IV 62, G. Halffter (1, INECOL); idem but VI.68, P. Reyes leg (1, INECOL); Chiapas, San Antonio Independencia 24-11-87, F. Arias (1); Chiapas, 35 mi N of Las Margaritas, 8 mi N of Leiva Velásquez, 28 VI 1986, J.C. Schuster, 1620m (2, UVGC); Chiapas, Rancho Santa Rosa, 11–17 1975, P. Hubbell (1, UVGC). GUATEMALA: Huehuetenango, Mnt. S of Yalambojoch, rd. to Bulej, 1800m, 5 IV 1996, E. Cano, cloud forest (11, UVGC); Huehuetenango, Nentón, montaña al sur de Yalambojoch, 22 VI 1996, bosque nuboso, P. Lucas, leg (51, UVGC); Huehuetenango, Nentón, Yalambojoch, 1800–1900m, 24 VII 1998, bosque nuboso, col. E.B. Cano (3, UVGC); Huehuetenango, Nentón, San Francisco, Fca. San Francisco, 21 sept 1998, C. Bailey, José Monzón (1, UVGC); Huehuetenango, Barillas, Laguna Maxbal, 27 VII 2000, B. nuboso, 1300m, E. Cano, tronco podrido (47, UVGC); Huehuetenango, Barillas, camino entre Nuevo San Mateo y San Juan las Milpas, cerca de Laguna Maxbal, 28–30 V 1998, E. Cano, bosque nuboso (11, UVGC); Huehuetenango, Barillas, Buena Vista Chiblac, 27 V 1998, E. Cano, b. nuboso (9, UVGC); Huehuetenango, Barillas, Nuevo San Mateo, 1200m, 15685062E, 1761883N, 27 mayo 1998, E.B. Cano, C. Bailey & J. Monzón (1, UVGC); Huehuetenango, Barillas, Malpais, 1200m, 15687633E, 1757386N, junio 1998, E.B. Cano, C. Bailey & J. Monzón (4, UVGC); Huehuetenango, Nentón, Bulej 2 km norte, bosque nuboso, 22–23 sept. 1998, C. Bailey, J. Monzón (2, UVGC); Huehuetenango, Barillas, aldea Malpais, 1100m, oct. 1998, S. Pérez, bosque nuboso (1, UVGC); Huehuetenango, 4 mi NW Barillas, Fca. Chiblac Buena Vista, 19–23 VII 1990, Brody + Campbell, 1300m, female (1, UVGC); Huehuetenango, Barillas, camino entre Ojo de Agua y la Laguna Maxbal, 27 VII 2000, b. nuboso, 1300m, E. Cano, en el suelo (4, UVGC); Huehuetenango Dept., ca 3km NW of Yalhuitz Grande, 1422m, 18–19 July 2012, N15°55.863', W91°17.960', R.S. Zack, coll. (1, UVGC); Huehuetenango, Barillas, Unión Las Palmas, 1444m, 15.9311000, -91.2993100, 28 mayo 2011, Camposeco & Monzón (5, UVGC); idem but 15 V 2012, b. nuboso, F. Camposeco, troncos (1, UVGC); idem but 29 VII 2011 (1, UVGC).

***Ogyges* sp. n. 2** (7). HONDURAS: Yoro, 10 km NO Morazán, 24 III 1991, J.Schuster, #WJe (2, UVGC); Parque Nac. Pico Pijol, Linda Vista, 1450m, N15°09', W87°37', 8–9 June 2007, RD Cave (1, RC); Pico Pijol, 3 June 2003, R. Turnbow (2, UVGC); Pico Pijol, 2 June 2003, R. Turnbow (2, UVGC).
